# Multiparametric Study of Antioxidant Effect on Ram Sperm Cryopreservation—From Field Trials to Research Bench

**DOI:** 10.3390/ani11020283

**Published:** 2021-01-23

**Authors:** Marta F. Riesco, Mercedes Alvarez, Luis Anel-Lopez, Marta Neila-Montero, Cristina Palacin-Martinez, Rafael Montes-Garrido, Juan Carlos Boixo, Paulino de Paz, Luis Anel

**Affiliations:** 1 Assisted Reproduction Techniques Research Group (Itra-ULE), INDEGSAL, University of León, 24071 León, Spain; mferrs@unileon.es (M.F.R.); mmalvg@unileon.es (M.A.); mneim@unileon.es (M.N.-M.); cpalm@unileon.es (C.P.-M.); rmong@unileon.es (R.M.-G.); juancarlos@boixo.com (J.C.B.); ppazc@unileon.es (P.d.P.); laner@unileon.es (L.A.); 2 Cell Biology, Department of Molecular Biology, University of León, 24071 León, Spain; 3 Animal Reproduction and Obstetrics, Department of Veterinary Medicine, Surgery and Anatomy, University of León, 24071 León, Spain; 4 Anatomy, Department of Veterinary Medicine, Surgery and Anatomy, University of León, 24071 León, Spain

**Keywords:** ovine, frozen sperm, antioxidants, fertility, sperm quality, redox balance

## Abstract

**Simple Summary:**

The use of antioxidant compounds could be a successful tool to improve sperm cryopreservation protocols in ovine species. These molecules have been widely employed in different mammalian species with this purpose. It is important to consider the existence of a species-specific antioxidant effect discarding the extrapolations from other animal species. To corroborate the real effectiveness of these compounds is important to combine two approaches: in vitro sperm quality analyses and in vivo field trials based on fertility. In the first scenario, a multiparametric analyses and novel tests based on spermatozoa redox balance, as the main target of antioxidants, could improve the accuracy on antioxidant effectiveness on sperm quality. Moreover, an extensive field insemination study provides the definitive tool to select the best antioxidant treatment. All these aspects have been applied and extensively discussed throughout this manuscript. Novel approaches have been incorporated, such as RedoxSYS, to provide more accuracy in the integrative studies of Redox status in spermatozoa. The effectiveness of an antioxidant treatment, as trolox in our study, should be demonstrated in an integrative way, from in vivo (fertility trials) to in vitro analyses (sperm quality assays), especially when the final aim is to reach AI implementation.

**Abstract:**

The optimization of sperm cryopreservation protocols in ram is a feasible tool to reinforce artificial insemination technologies considering the desirable application of sperm by vaginal/cervical or transcervical deposition. Cryopreservation provokes different types of damage on spermatozoa and many of these detrimental effects are triggered by redox deregulation. For this reason, the antioxidant supplementation in sperm cryopreservation protocols to decrease reactive oxygen species (ROS) levels and to equilibrate redox status has been widely employed in different species. Despite this, more fertility trials are necessary to provide the definitive tool to ensure the antioxidant effectiveness on sperm quality. For this reason, in this work, we performed a multiparametric analysis of some previously tested antioxidants (crocin, GSH and Trolox) on ram sperm cryopreservation from field trials to sperm quality analyses focused on new strategies to measure redox balance. Attending to fertility trial, Trolox supplementation registered an improvement concerning to fertility (when we considered high fertility males) and multiple lambing frequency and other complementary and descriptive data related to lambing performance such as prolificacy and fecundity. This positive effect was more evident in multiple lambing frequency when we considered low fertility males than in global male analysis. In vitro analyses of sperm quality confirmed in vivo trials registering a positive effect on sperm viability and redox balance. In this study, we provided the definitive evidence that the role of trolox on redox balance maintenance has a direct effect on fertility parameters, such as prolificacy. The effectiveness of antioxidant treatments was tested, for the first time in ovine species, using an integrative and multiparametric approach combining in vivo and in vitro analyses and novel approaches, such as RedoxSYS. These types of strategies should be applied to improve sperm conservation methods and optimize AI technologies upgrading the correlation between in vitro and in vivo analyses.

## 1. Introduction

Sperm cryopreservation is an important tool in artificial reproductive technologies (ART), selection of animals with superior genetic traits, clinical medicine and perpetuation of species [1,2]. In the same way, this technique preserves the genetic variability of wild populations and allows to storage material of breeds at risk of extinction. Different artificial insemination (AI) methods have been described according to semen deposition in ovine species and sperm storage method—by vaginal deposition of semen, cervical (intracervical deposition of semen) or intrauterine (laparoscopic technique) deposition [2,3]. Although successful fertility rates have been reached using frozen sperm by laparoscopic method in ovine species, the results obtained with other methods for sperm deposition have been less hopeful. In these IA alternative methods, the optimization of sperm cryopreservation is a mandatory question [4]. An improvement in sperm quality could be crucial to establish alternative methodologies for laparoscopic technology that could simplify the AI techniques.

In general, sperm cryopreservation provokes a high production of reactive oxygen species (ROS) triggering different level of cryodamage that compromises sperm quality and fertility [5,6,7]. In the beginning, oxidative stress was just characterized as overproduction of ROS; however, nowadays it is known as a consequence of redox deregulation. The imbalance between ROS levels and physiologic antioxidant levels can trigger oxidative stress responses [8]. Spermatozoa are highly susceptible to the deleterious effects of ROS due to the large amounts of unsaturated fatty acids found in their cell membranes [9]. It is widely known that increased ROS levels involve lipid peroxidation [10], loss of membrane integrity with increased permeability triggering detrimental consequences on sperm quality such as: a decrease in sperm motility, DNA fragmentation, apoptosis and decreased oocyte-sperm fusion [11,12,13].

Pro- and anti-oxidant systems are normally presented in a balanced state to modulate the ROS levels controlling different biological functions and preventing pathological processes [14,15]. Antioxidants are capable of counteracting ROS overproduction during sperm cryopreservation; however, when ROS generation overwhelms the antioxidants’ threshold of ROS clearance or when antioxidant production is diminished, a state of oxidative stress ensues [16]. The antioxidant content of sperm is limited and can be reduced by extender dilution during the preservation procedures decreasing its beneficial effect on sperm quality [17]. Thus, antioxidant supplementation, even in low concentrations, during sperm cryopreservation emerges as a promising tool to optimize these types of procedures. The possible beneficial effects of these antioxidants on sperm quality during cryopreservation technologies have been characterized and widely reviewed in different species such as human, boar, red deer or ram among others [17,18,19,20,21,22,23,24]. However, there is no information about the effectiveness of these antioxidant treatments on reproductive outcomes after AI. In addition, there is a lack of consistency between ram sperm quality analyses and fertility success in many situations [25]. Likely, molecular alterations during freezing procedures may account for such low consistency. New approaches to detect them are peremptory, since they might be still undetectable. In this sense, nowadays, new integrative strategies based on redox balance, such as RedoxSYS system, have been implemented in spermatology analyses [11]. RedoxSYS index, specially sORP, has been negatively correlated to fertility in different species [26]. This new approach could contribute to improve the knowledge of sperm cryodamage and its relationship with fertility success.

In a previous study performed by our group, different antioxidants (crocin, GSH, cysteamine and trolox) were evaluated during ram sperm cryopreservation [27]. These antioxidants were selected by their potential beneficial roles preserving sperm quality during freezing and thawing procedures. In this work, the authors can conclude that Trolox supplementation had positive effects on sperm viability and reduced malondialdehyde production provoking a decrease in lipid peroxidation [27]. Considering such promising results, the aim of this work was to test if these effects of antioxidant supplementation on sperm quality could be translated in fertility upgrading, with the aim to obtain a total confirmation of their effectiveness in an integrative way.

## 2. Materials and Methods

### 2.1. Ethics Statement

The current study was performed according to the Guidelines of the European Union Council (86/609/EU, modified by 2010/62/EU), following Spanish regulations (RD/1201/2005, abrogated by RD/2013) for the use of laboratory animals. All experimental protocols and procedures were approved by the institutional Animal Care and Use Committee at the University of León (Spain) (ETICA-ULE-034-2020).

### 2.2. Sample Collection

Adult Churra rams of proven fertility aged between two and five years were the subjects of the experiments. Ram ejaculate was obtained from trained males (weekly semen collections, twice a week) during the breeding season (autumn) in Ovigén (Centro de Selección y Mejora Genética de Ovino y Caprino, Toro, Junta de Castilla y León, Spain). The ejaculate was collected by artificial vagina (water at 40 °C) and the sample tubes were kept in a water bath at 35 °C during the initial evaluation of semen quality. Volume was measured and determined with a graduated tube of polystyrene. Mass motility was assessed with a subjective score of 0–5 by a microscope equipped with a warmed stage programmed at 37 °C (Leica DM LB, Meyer Instruments, Houston, TX, USA) and a bright field objective −4x. Sperm concentration was analyzed by the photocolorimetric method at 540 nm, using a specifically calibrated scale. Only ejaculates of good quality were used (volume ≥ 0.5 mL, mass motility ≥ 3 and concentration ≥ 3 × 10^9^ sperm/mL).

### 2.3. Experimental Design and Antioxidant Supplementation

Ejaculates were processed according to Anel and colleagues [28]. Briefly, the samples were diluted with the same volume (1:1) of TES-Tris-fructose media (TES 224 mM, Tris 85 mM, fructose 13 mM, adding 10% clarified egg yolk and 4% glycerol, 320 mOsm/kg, pH 7.2; UL extender) and cooled down to 5 °C. Then, each ejaculate was split and extended to 100 × 10^6^ of spermatozoa/mL with the same base extender in four subsamples to obtain the experimental groups: control (without antioxidant supplementation) and three groups whose extender was supplemented with antioxidants at 1 mM of GSH, 1 mM of crocin and 1 mM of trolox. In the present study, frozen-thawed doses from seventeen males were employed: nine males were selected for the fertility tests and eight different males were used in sperm quality analyses.

### 2.4. Freezing and Thawing Protocols

Sperm samples were frozen in 0.25 mL French straws (IMV Technologies, L’Aigle, France), using a programmable biofreezer (Kryo 10 Series III; Planer PLC, Sunbury-on-Thames, UK) at a rate of −20 °C/min down to −100 °C. Then, the straws (10 × 10^7^ of spermatozoa/mL) were kept in liquid nitrogen containers for at least a month. Straws were thawed in a water bath at 65 °C for 6 s.

### 2.5. Fertility Trial: Field Inseminations

For the fertility trial, 562 sperm doses (25 × 10^6^ sperm/dose) from nine mature males (Churra breed), were thawed in a water bath at 65 °C for 6 s. The samples were randomly (per male) and sequentially (per experimental group) distributed through 10 commercial farms following a commercial artificial insemination program (Churra breed improvement program) under the strict supervision of our research group. Adult Churra ewes (562 females) were subjected to treatment for estrous induction and synchronization using intravaginal sponges with 20 mg fluorogestone acetate (Chronogest^®^, MSD, Kenilworth, NJ, USA, EEUU) over 14 days. The sponges were removed and 500 IU of eCG were intramuscular (i.m.) injected (Folligon^®^, MSD, Bogotá, Colombia). Laparoscopic inseminations were performed by two experienced veterinarians of our research group, between 62 and 64 h after the removal of the sponges. The animals, having fasted for the previous 24 h, were tied to a special cradle (IMV, L’Aigle, France), placed on an inclined plane (positive Trendelenburg, 45°) and the area in front of the teat was shaved and cleaned. Local anesthesia (mepivacaine HCL 2%, BraunTM, Barcelona, Spain) was applied to the puncture points. Then, two portals (for vision and manipulation/injection) were inserted by performing a pneumoperitoneum (CO_2_). The semen, placed in a special applicator (Transcap^®^, IMV, L’Aigle, France), was injected under visual inspection into each uterine horn (0.12 mL, 12.5 × 10^6^ spermatozoa). Reproductive success was evaluated in terms of fertility (lambing ewes according to the births registered at 137–154 days post-insemination). Viable offspring, sex ratio and lambing performance (specifically multiple lambing frequency) were registered. Additionally, some complementary descriptive data of lambing performance were calculated: prolificacy (lambs/lambed ewe), fecundity (lambs/inseminated ewe) and litter size distribution (See supplementary results in Appendix A, Table A1).

### 2.6. In Vitro Assay: Sperm Quality Analyses

A total of eight sperm samples (one per male) were analyzed in each experimental group.

#### 2.6.1. Sperm Motility and Kinetic Parameters

Sperm motility and kinetics were performed using computed assisted sperm analysis (Sperm Class Analyzer (SCA) software v. 6.3.0.59 (Microptic S.L., Barcelona, Spain). Sperm samples of the different experimental groups were diluted to obtain a final concentration of 2 × 10^7^ sperm/mL in TES-Tris-fructose media supplemented with 1% clarified egg yolk (pH = 7.5, 300 mOsm/kg) and warmed to 37 °C on a warmed plate. Five microliters of the diluted semen of each experimental group were dropped onto a Makler counting chamber (10 µm depth; Sefi Medical Instruments, Mumbai, India) and analyzed with SCA. The SCA system consisted of an optical phase-contrast Nikon Eclipse microscope (Nikon, Tokyo, Japan) equipped with a Basler A312fc digital camera (Basler Vision Technologies, Ahrensburg, Germany) with a warmed stage (37 °C), using a 10× objective with negative phase contrast specifically set for ram spermatozoa (1 µm^2^ < particle area <20 µm^2^). The sperm quality parameters included in our study were: the percentage of total motile spermatozoa (TM, %) defined as the percentage of sperm with VCL >15 μm/s, progressive motility (PM) defined as the percentage of sperm with VCL >45 μm/s and straightness (STR) >80% and fast progressive motility population fast progressive motility (FPM) defined as the percentage of sperm with VCL >75 μm/s.

#### 2.6.2. Multiparametric Flow Cytometry Analyses

Sperm samples of different experimental groups were diluted in phosphate-buffered saline (PBS) medium to obtain a total of 2 × 10^6^ of spermatozoa per sample; these samples were centrifuged at 500× *g* for 10 min at RT. The flow cytometry staining protocol has been previously described by Riesco et al., 2020 [29]. Briefly, the supernatant was discarded and the sperm pellet was incubated at RT for 30 min in the dark with 96 μL of Zombie Violet™ (membrane integrity probe) (1:1000 final dilution in PBS, Biolegend, Madrid, Spain), 2 μL of CellEvent™ Caspase-3/7 (apoptosis marker) (4 μM final concentration in PBS, Thermo Fisher, Madrid, Spain) and 2 μL of CellROX^®^ (ROS content labeling) (5 μM final concentration in PBS, Thermo Fisher, Madrid, Spain). After that, another washing step was performed to prevent cell staining and the pellet was resuspended in 1 mL of PBS, carrying out the analysis immediately by flow cytometry.

Flow cytometry acquisition was performed in a flow cytometer (MACSQuant Analyser 10, Miltenyi Biotech, Madrid, Spain) equipped with three lasers emitting at 405 nm, 488 nm and 635 nm and 10 photomultiplier tubes (V1 (excitation 405 nm, emission 450/50 nm), V2 (excitation 405 nm, emission 525/50 nm), B1 (excitation 488 nm, emission 525/50 nm), B2 (excitation 488 nm, emission 585/40 nm), B3 (excitation 488 nm, emission 655–730 nm (655 LP + split 730), B4 (excitation 499 nm, emission 750 LP), R1 (excitation 635 nm, emission 655–730 nm (655 LP + split 730) and R2 (excitation 635 nm, emission filter 750 LP). The system was controlled using MACS Quantify software (Miltenyi Biotech, Madrid, Spain). These excitation and emission wavelengths allowed us to find probe combinations that can simultaneously assess multiple parameters in a large number of spermatozoa (10,000 sperm cells). Data were analyzed using FlowJo v.10.2 (Ashland, Wilmington, DE, USA).

#### 2.6.3. RedoxSYS Analysis

Oxidation reduction potential is a measure of the transfer of electrons from a reductant (or antioxidant) to an oxidant and was measured by a galvanostatic-based technology, the RedoxSYS assessment (Luoxis Diagnostics, Englewood, CO, USA). This diagnostic system provides two values: (i) sORP, the integrated balance of oxidants and reductants in a specimen, reported in millivolts (mV); and (ii) cORP, the amount of antioxidant reserves, expressed in microcoulombs (μC). In particular, sperm samples of the experimental groups (control, GSH, crocin and trolox) were washed two times with PBS 1X to remove cryoprotectant, extender and antioxidant compounds. After that, 20 μL of sperm samples (1 × 10^6^ sperm cells) were applied to disposable sensors designed by Luoxis, which were inserted into the RedoxSYS diagnostic system, which measured and reported within 4 min the sORP and cORP values. Measures were registered in triplicate for each sample. The average values for sORP and cORP were recorded. Finally, these data were presented as mV/10^6^ sperm for sORP and μC/10^6^ sperm for cORP.

### 2.7. Statistical Analysis

All statistical procedures were performed using SAS v9.1 (SAS/STAT; SAS Institute Inc., Cary, NC, USA). In reproductive success studies, antioxidants were tested for effects on fertility (yes/no), sex ratio (male/female) and lambing performance (single/multiple) [30,31]. Models included the random effect of sperm dose. Binary response variables were analyzed using the GENMOD procedure. A total of 562 inseminations were analyzed. To identify changes in sex ratio distortion (female-biased or male-biased) from the reference value (50:50) [32,33], the Student’s t-test (μ = 50, 50:50 ratio) was performed after Bonferroni-Holm multiple testing correction in antioxidant supplementation treatments. Significant differences were considered at *p* < 0.05. Finally, we carried out and independent statistical analyses considering two additional classes according to the obtained fertility data of this study: high fertility males (HFM) and low fertility males (LFM). These analyses was performed to investigate a possible different response after antioxidant supplementation considering these two additional groups. Concerning to in vitro sperm analyses, data were analyzed using a mixed linear model (MIXED procedure). The normality of variables was examined. The antioxidants were included as the main effects and the male was recorded as random effects in the model. The differences between LS-means (Least Squares Means) were computed by Dunnett’s test. Results are displayed as means ± SEM (Standard Error of the Mean). A total of eight individual males were analyzed in each experimental group. Significant differences were considered at *p* < 0.05. In the case of sperm quality tests, the statistical analysis based on fertility groups (HFM and LFM) was not considered due to absence of fertility data of these males.

## 3. Results

### 3.1. In Vivo Results: Fertility Trials

**Figure 1:**
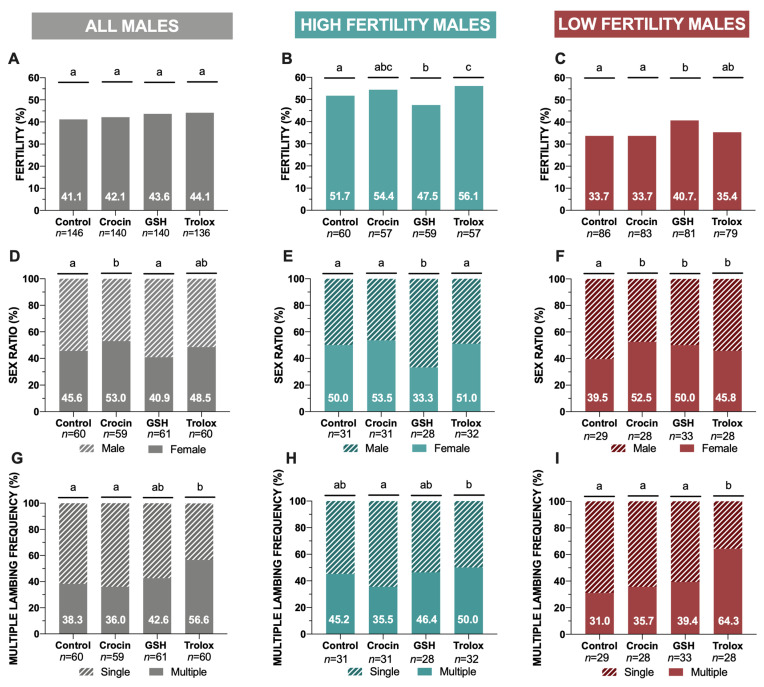
Ram cryopreserved sperm fertility trials in the four experimental groups (control: without antioxidants, 1 mM GSH, 1 mM crocin and 1 mM trolox supplementation). (**A**) Global fertility (lambed ewes/inseminated ewes, %) including all analyzed males; (**B**) Fertility (%) in high fertility males (HFM); (**C**) Fertility (%) in low fertility males (LFM); (**D**) Global sex ratio results (females vs. males frequency, %) including all analyzed males; (**E**) sex ratio analysis (lambed ewes/inseminated ewes) (%) in high fertility males (HFM); (**F**) sex ratio analysis (lambed ewes/inseminated ewes) (%) in low fertility males (LFM); (**G**) Lambing performance (multiple lambing frequency, %) considering all males; (**H**) Multiple lambing frequency (%) in high fertility males (HFM); (**I**) Multiple lambing frequency (%) in low fertility males (LFM). Significant differences (*p* < 0.05) among experimental groups are represented with different lowercase letters. A total number of 562 inseminated females were analyzed were employed in the fertility trials.

Concerning to fertility (lambed ewes/inseminated ewes, %), when it was considered all males, significant differences were not found among antioxidant treatments in relation to the control (without antioxidants) (Figure 1A). However, only considering high fertility males (HFM) and low fertility males (LFM) in an independent analysis, in the HFM group, trolox supplementation resulted in a higher percentage (*p* < 0.05) of fertility in comparison to the control group (Figure 1B). For its part, in the LFM, GSH supplementation increased (*p* < 0.05) the percentage of lambed ewes (per inseminated ewes) in relation to the control group and the crocin treatment (Figure 1C).

When we attend to sex ratio considering all males, crocin treatment showed a significant increase (*p* < 0.05) in female frequency in relation to the control (Figure 1D). In HFM group, GSH treatment provided the lowest female frequency (*p* < 0.05) among treatments (Figure 1E). In contrast, for LFM group, the antioxidant supplementation provoked higher (*p* < 0.05) female proportion in the offspring comparing to the control group, regardless of the antioxidant used (Figure 1F).

In terms of lambing performance, trolox supplementation significantly increased (*p* < 0.05) the percentage of multiple lambing in comparison to the samples without antioxidants (Figure 1G). Also, this treatment registered the highest values of prolificacy (lambs/lambed ewe) (1.62 ± 0.08) and fecundity (0.71 ± 0.08) comparing to the control without antioxidant supplementation (1.42 ± 0.07 and 0.58 ± 0.07) and with other antioxidant treatments (Appendix A, Table A1). However, when we analyzed HFM and LFM independently, trolox registered a higher effectivity in LFM than in HFM concerning the frequency of multiple lambing (Figure 1H,I).

### 3.2. In Vitro Results: Sperm Quality Assays

**Figure 2:**
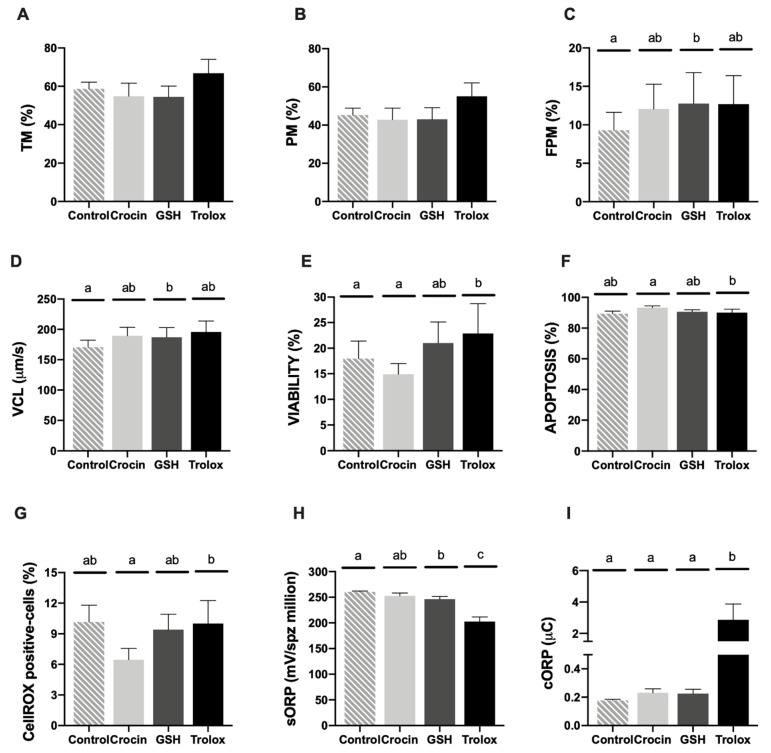
Ram cryopreserved sperm motility and multiparametric flow cytometry analyses in the four experimental groups (control: without antioxidants, 1 mM GSH, 1 mM crocin and 1 mM trolox supplementation). (**A**) Total motility (TM, %); (**B**) progressive motility (PM, %); (**C**) fast progressive motility (FPM, %); (**D**) curvilinear velocity (um/s); (**E**) Zombie negative cells (viability, %); (**F**) caspase 3/7 positive cells (apoptosis, %); (**G**) CellROX-positive cells (sperm ROS levels, %); (**H**) static ORP (sORP) index (mV/10^6^ sperm); (**I**) capacitance ORP (cORP) index (μC/10^6^ sperm). Results are expressed as the mean ± S.E.M. A total of eight males were analyzed in each experimental group. Significant differences (*p* < 0.05) are represented with different lowercase letters.

**Figure 3:**
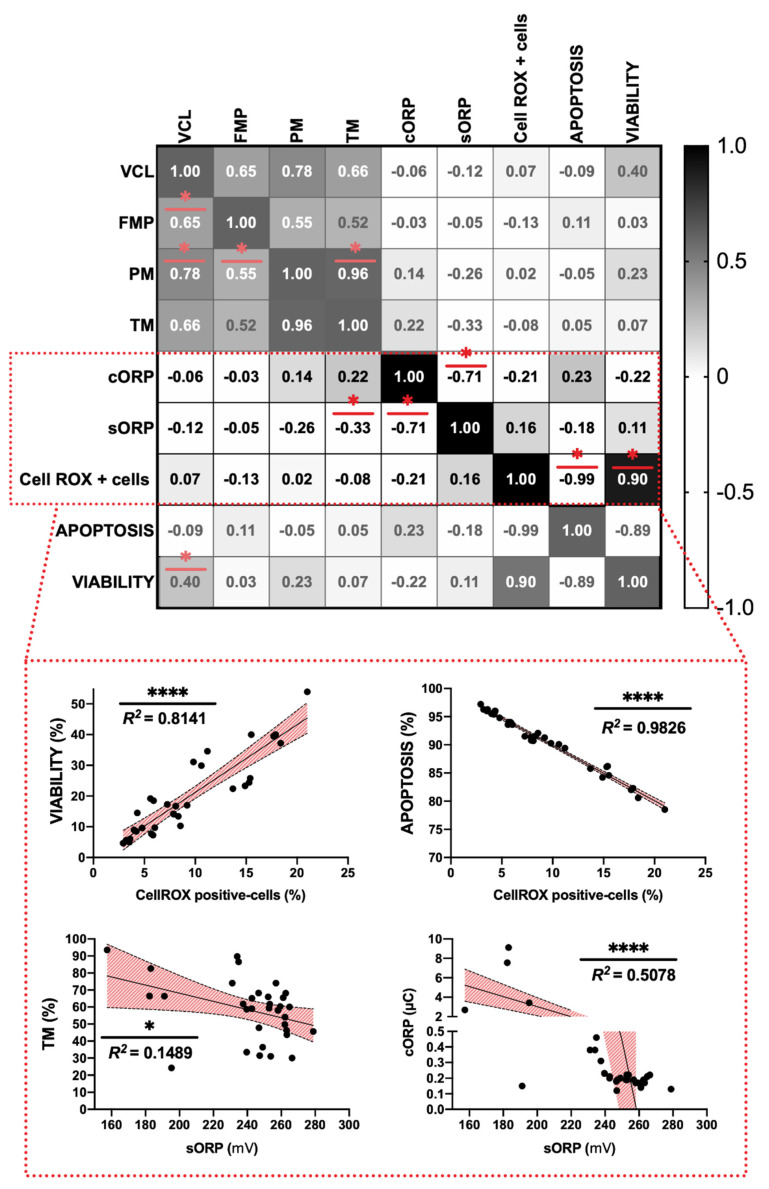
Correlation matrix of all sperm quality markers studied and individual significant correlations between some parameters. The experimental groups are included to calculate correlation matrix individual correlations. The R squared value between two parameters is represented in each cell and graph. In the correlation matrix, dark color indicates positive correlations and clear color indicates negative relationships. The color intensity represents the strength of the correlation between two sperm quality parameters. Asterisks show significant correlations (*p* < 0.05) among in vitro sperm quality parameters. The number of asterisks (*) indicates the significance levels: one asterisk (*) indicates *p* < 0.05 and four asterisks (****) indicate *p* < 0.0001.

Motility parameters (TM and PM) did not register significant differences among treatments (Figure 2A,B). Concerning FPM, GSH treatment significantly (*p* < 0.05) ameliorated this population with respect to the thawed control without antioxidant supplementation (Figure 2C). The same pattern was observed regarding to VCL (Figure 2D). Attending to the flow cytometry analyses, the addition of trolox provided the better results of sperm viability (*p* < 0.05) comparing to control (Figure 3E). This compound showed a significant decrease of apoptotic sperm (Figure 2F) accompanied with a significant increase in ROS content (Figure 2G) in comparison to crocin supplementation.

Concerning to RedoxSYS, the addition of trolox registered the lowest sORP value and the highest cORP indexes among treatments (*p* < 0.05) (Figure 3H,I). Moreover, GSH antioxidant improved sORP index comparing to the control without antioxidant (*p* < 0.05) (Figure 2G).

### 3.3. Correlation of Redox Balance Parameters

Among sperm analyses, the highest strength of negative correlation was found between ROS content and apoptosis incidence (R^2^ = 0.9826) and between Cell-ROX positive-cells and viability (R^2^ = 0.8141) (*p* < 0.05) (Figure 3). ROS content was significantly correlated with viability and apoptosis parameters (Figure 3). Attending to RedoxSYS parameters, sORP index presented a significant and negative correlation with total motility (TM) (R^2^ = 0.1489) and also with cORP index registering values around R^2^ = 0.71 (Figure 3).

## 4. Discussion

The optimization of sperm conservation methods to achieve an improvement in sperm management are the most feasible alternative to standardized more demanding artificial insemination (AI) techniques such as vaginal deposition in ovine species [2,3]. In this sense, the maintenance of redox balance represents one of the most important challenges to overcome the cryodamage during sperm conservation methods. Increased ROS along with decreased antioxidant defense result in a redox imbalance. It is widely known that a Redox deregulation trigger detrimental effects on sperm quality mainly related to reduced sperm motility, structural DNA damage and apoptosis [10,18,34]. Therefore, antioxidant supplementation had been widely employed in different species to counteract when the ROS concentration exceeds the physiological limit during sperm cryopreservation technologies [15,16]. There is a high number of evidence to indicate that antioxidants can reverse the negative impact of oxidative stress sustained during sperm cryopreservation in a recent large number of disparate species including the stallion [35], boar [36], bull [37], goat [38] or red deer [39] among others [7]. Specifically, in ram sperm, a variety of studies have been performed to diminish cryodamage adding antioxidant agents and extensively reviewed by Allai and colleagues [17]. However, there are scarce field fertility trials confirming the antioxidant beneficial effects on sperm quality during freezing and thawing procedures. Under our knowledge, this is the first time in sheep demonstrating the beneficial effects of antioxidant supplementation during sperm cryopreservation in an integrative way: from field inseminations to in vitro sperm quality analyses. Considering our previous published results [27] based on the beneficial effect on ram thawed sperm quality of antioxidant addition, in a first step we decided to perform the fertility trial to provide the definitive probe of trolox effectiveness. We observed previously that trolox improved sperm quality and with the current work confirmed this positive effect of trolox addition attending to multiple lambing frequency (Figure 1G). Concerning to fertility results, Trolox manifested its positive effect only in HFM group (Figure 1B). With GSH supplementation (1 mM), some similar tendencies with a less noticeable effect were observed that ameliorated (*p* < 0.05) fertility efficiency in the LFM group comparing to the control without antioxidants (Figure 1C). The different response observed in HFM and LFM to the specific antioxidants could be associated with the specific target of sperm parameters where the antioxidant is acting. Concerning to sex ratio index, all the antioxidants under study increased the female percentage in the LFM comparing to the control sperm samples (Figure 1F). The relationship between sperm quality and sex ratio is a controversial concern and this fact has been previously studied in different mammalian species including human sperm [40]. Thus, while some studies found positive correlations between sperm speed and sex ratio to male offspring [41], others have reported that between sperm motility and higher female births [42]. However, some studies performed in mice concurred that differences in sperm quality (DNA damage) could provoke sex ratio distortion from the expected value [33]. These authors claimed that this altered environment with high levels of ROS affected the functionality of sperm carrying X or Y chromosomes [33,43]. This distortion can be trigger by an increased level of oxidative stress in the spermatozoa environment that it has been previously suggested. According to these findings, the process of cryopreservation itself and antioxidant addition that counteracts oxidative stress could directly affect sex ratio. In our study, we tested through statistical analyses that our data did not present distortion from 50:50 ratio (reference value) [44] after antioxidant supplementation and cryopreservation discarding an effect of these compounds on this parameter.

Sperm motility and kinetics analyses revealed a slight upgrading on some parameters (FPM and VCL) after GSH treatment in respect to the thawed control (Figure 2A–D). Trolox supplementation registered an increase in sperm viability (Figure 2E). These results confirmed our previous study, where trolox considerably reduced oxidative stress through reducing malondialdehyde production (lipid peroxidation) and improving sperm membrane integrity [27]. Lipids are responsible for the fluidity of sperm membrane layers. The plasma membrane of mammalian spermatozoa contains high levels of lipids in the form of polyunsaturated fatty acids (PUFAs). These lipids contain unconjugated double bonds separated by methylene groups making hydrogen extremely prone to oxidative damage. When the levels of ROS within the cell are high, ROS will attack these PUFA, causing a disruption in membrane integrity [15]. The correlation between sperm quality and reproductive success has been widely discussed [25,45]. However, some studies performed in ram confirmed the hypothesis that spermatozoa of high fertility rams displayed higher viability than low fertility rams [45,46]. This fact could be explained attending to the plasma membrane of sperm is essential for metabolic functions, capacitation, zona pellucida binding, acrosome reaction and membranes fusing. Hence, the loss of integrity of this structure is considered incompatible with sperm fertilization capacity [47]. It is important to remark, that the present study did not reveal significant differences concerning to ROS content among treatments (Figure 2). Moreover, when we observed the CellROX staining tendency we detected that the treatments that reported higher viability presented more percentage of CellROX positive-cells than the treatments that registered lower viable sperm cells (Figure 2E,G). This statement was confirmed by the correlation matrix results, where we observed a significant positive correlation between ROS content and viability and contrary to this, a negative high strength of correlation was detected between this ROS-related probe and apoptotic sperm population (*p* < 0.005) (Figure 3). This fact could be explained by attending to previous findings in stallion sperm that demonstrated, that the CellROX probe mainly reflects intense mitochondrial activity rather than oxidative stress [48]. For these reasons, this assay firstly designed to detect ROS content that could be feasible as biomarker in antioxidant supplementation studies should be replace for other novel analysis that reflect in an accurate way the redox balance status.

A novel assay based on Redox status, RedoxSYS, providing the overall oxidant and antioxidant activity was performed. Traditional analyses related to redox balance are focused on chemiluminescence for ROS, total antioxidant capacity for antioxidants and the malondialdehyde assay to detect lipid peroxidation [49]. Additionally, assay results do not correlate with one another and provide only a single marker of oxidative stress -either oxidant levels, antioxidant levels or triggered damage [34]. RedoxSYS technology allows to measure the overall oxidant and antioxidant activity in a given sperm sample providing a more realistic picture of redox status [50]. This assay was revealed as the most accurate test, specially sORP index, according to fertility trial results, revealing GSH and trolox as the best antioxidant agents in terms of Redox status maintenance (Figure 2H,I). In contrast, no significant differences were found concerning to apoptosis incidence among treatments (excepting between crocin and trolox, due to the negative effect of the crocin) (Figure 2E). It has been demonstrated that moderate ROS levels that are not sufficient to induce apoptosis are still capable of disrupting some aspects of sperm functionality such as motility and sperm oocyte fusion [14]. In addition, sublethal levels of oxidative stress are known to impact the integrity of sperm DNA thereby influencing fertility trials concerning to development of the embryo [14,51]. More importantly, these parameters could be more reliable indicators of the reproductive success due to their non-compensable character [25]. After oocyte penetration, the paternal contribution depends on completely on the quality of the spermatozoon; specifically of intrinsic sperm quality factors such as DNA and chromatin status. These intrinsic factors determine the maintenance of the fertilization process and subsequent embryogenesis (once initiated). On the other hand, other sperm parameters related to morphological and functional traits such as sperm motility, membrane integrity, acrosome, viability and so forth are considered compensable factors [52]. These compensable factors determine that sperm reaches the fertilization site and initiates the egg activation process and can be overcome by increasing the number of sperm inseminated on the contrary to non-compensable factors that cannot be overcome [53,54,55]. This fact provokes that the non-compensable factors contribute most to the fertility rates and other parameters such as lambing performance (multiple lambing frequency, prolificacy and fecundity). Considering ROS production as the main factor promoting DNA fragmentation during cryopreservation protocols [14,56] antioxidant supplementation could offer a target response to avoid DNA damage. In this scenario, sperm quality analyses focus on redox balance that trigger DNA damage can be considered as an interesting and predictive approach in these studies.

To summarize, trolox supplementation (1 mM) represented the best antioxidant agent combined the highest number of positive effects on fertility trials and the best sperm quality analyses although GSH treatment presented some beneficial effects too. In the case of trolox, the same positive effect was registered when we provided complementary descriptive data concerning to lambing performance with high interest in ovine exploitations such as prolificacy and fecundity (Appendix A, Table A1). These obtained results are according to previous ones in different mammalian species including human species [27,57,58,59,60]. The role of this Vitamin E analog on lipid peroxidation, membrane integrity and motility has been demonstrated in different studies on sperm cryopreservation [27,57,58,59,60]. Contrary to our results, some research works in ram and red deer sperm have claimed that trolox effect could be detrimental for sperm functionality and this dual effect could be dose-dependent, this fact has been recently reviewed [17,39]. However, in this study we provided the definitive evidence that the role of trolox on redox balance maintenance has a direct effect on reproductive results of AI, such as lambing performance (multiple lambing frequency Figure 1G; prolificacy and fecundity Appendix A, Table A1).

## 5. Conclusions

1 mM trolox supplementation in cryopreservation extender represents an interesting approach to improve thawed sperm quality decreasing cryodamage in ovine sperm and increasing the reproductive performance in the fertility trials. This type of strategies could be a step-forward to spread and implement AI procedures in ovine species.

## Data Availability

The data presented in this study are available on request from the corresponding author.

## Appendix A

**Table A1:** Additional fertility parameters obtained after insemination with ram cryopreserved sperm in the four experimental groups (Control-without antioxidant, crocin, GSH and trolox): Prolificacy (Lambs/lambed ewe), Fecundity (Lambs/inseminated ewe) and Litter size distribution. Each table represents global, high fertility males (HFM) and low fertility males (LFM) data.

Global	Prolificacy	Fecundity	Litter Size Distribution			
			1	2		3
Control	1.42 ± 0.07	0.58 ± 0.07	61.8	35.0		3.3
Crocin	1.41 ± 0.08	0.59 ± 0.07	64.4	30.5		5.1
GSH	1.44 ± 0.07	0.63 ± 0.07	57.4	41.0		1.6
Trolox	1.62 ± 0.08	0.71 ± 0.08	43.3	51.7		5.0
**HFM**	**Prolificacy**	**Fecundity**	**Litter Size Distribution**			
			**1**	**2**		**3**
Control	1.52 ± 0.11	0.78 ± 0.11	69.0	31.0		0
Crocin	1.39 ± 0.10	0.75 ± 0.11	64.3	28.6		7.1
GSH	1.50 ± 0.11	0.71 ± 0.11	60.6	39.4		0
Trolox	1.53 ± 0.10	0.86 ± 0.12	35.7	57.1		7.1
**LFM**	**Prolificacy**	**Fecundity**	**Litter Size Distribution**			
			**1**	**2**	**3**	
Control	1.31 ± 0.09	0.44 ± 0.07	54.8	38.7	6.5	
Crocin	1.43 ± 0.12	0.48 ± 0.08	64.5	32.3	3.2	
GSH	1.39 ± 0.09	0.57 ± 0.08	53.8	42.9	3.6	
Trolox	1.71 ± 0.11	0.61 ± 0.10	50.0	46.9	3.1	
